# Physiological oxygen culture reveals retention of metabolic memory in human induced pluripotent stem cells

**DOI:** 10.1371/journal.pone.0193949

**Published:** 2018-03-15

**Authors:** Alexandra J. Harvey, Carmel O’Brien, Jack Lambshead, John R. Sheedy, Joy Rathjen, Andrew L. Laslett, David K. Gardner

**Affiliations:** 1 School of BioSciences, University of Melbourne, Parkville, Victoria, Australia; 2 ARC Special Research Initiative, Stem Cells Australia, Melbourne, Victoria, Australia; 3 CSIRO Manufacturing, and Australian Regenerative Medicine Institute, Monash University, Clayton, Victoria, Australia; 4 School of Medicine, University of Tasmania, Hobart, Tasmania, Australia; University of Texas at Austin Dell Medical School, UNITED STATES

## Abstract

Reprogramming somatic cells to a pluripotent cell state (induced Pluripotent Stem (iPS) cells) requires reprogramming of metabolism to support cell proliferation and pluripotency, most notably changes in carbohydrate turnover that reflect a shift from oxidative to glycolytic metabolism. Some aspects of iPS cell metabolism differ from embryonic stem (ES) cells, which may reflect a parental cell memory, or be a consequence of the reprogramming process. In this study, we compared the metabolism of 3 human iPS cell lines to assess the fidelity of metabolic reprogramming. When challenged with reduced oxygen concentration, ES cells have been shown to modulate carbohydrate use in a predictably way. In the same model, 2 of 3 iPS cell lines failed to regulate carbohydrate metabolism. Oxygen is a well-characterized regulator of cell function and embryo viability, and an inability of iPS cells to modulate metabolism in response to oxygen may indicate poor metabolic fidelity. As metabolism is linked to the regulation of the epigenome, assessment of metabolic responses of iPS cells to physiological stimuli during characterization is warranted to ensure complete cell reprogramming and as a measure of cell quality.

## Introduction

Reprogramming of somatic cells to pluripotency is associated not only with the remodelling of nuclear architecture, epigenetics and gene expression but also with the reprogramming of metabolism. Significantly, changes to metabolism precede the up-regulation of pluripotent gene expression and constitute one of the earliest events in induced pluripotent stem (iPS) cell formation [1, 2]. Manipulation of metabolism during somatic cell reprogramming impacts reprogramming efficiency, highlighting the importance of metabolic change to the process. Reprogramming is enhanced by agents that promote glycolysis [2, 3], or by culture under physiological oxygen conditions [4], while inhibition of glycolysis impairs iPS reprogramming [2, 3]. Like embryo-derived embryonic stem (ES) cells, successfully reprogrammed iPS cells show a dependence on glycolysis for ATP production, and significantly higher lactate production, when compared to either fibroblasts or their somatic progenitors [5, 6]. Total cellular ATP [2, 7, 8], oxygen consumption [2, 8], mitochondrial mass [9] and mitochondrial DNA (mtDNA) copy number [10, 11], are reprogrammed to more ES cell-like levels in mouse and human iPS cells, while genes regulating glycolysis, the Pentose Phosphate Pathway (PPP), the TCA cycle, and mitochondrial complex activity are also altered to levels similar to that of ES cells [1, 2, 8, 11]. These changes demonstrate the occurrence of a shift in metabolism during reprogramming to a pluripotent cell state and underscore the importance of metabolism in the acquisition and maintenance of pluripotency.

Investigating the fidelity of reprogramming to pluripotency has suggested that some iPS cell lines retain a somatic transcriptional and epigenetic memory [12, 13] and, for virally transfected lines, a propensity to revert to a pluripotent phenotype following short-term differentiation [14]. In addition, several reports have demonstrated that some metabolic pathways are not reliably reprogrammed to ES-cell like levels during iPS cell formation. Human iPS cells characteristically show lower levels of unsaturated fatty acid metabolites and increased levels of metabolites involved in the s-adenosyl methionine (SAM) cycle when compared to ES cells [15]. Several studies have concluded that reprogramming is associated with a complete remodelling of mitochondria to a pluripotent state in iPS cells. However, transmission electron micrographs show a proportion of mitochondria in mouse and human iPS cells which retain a cristae-rich, elongated architecture [2, 8, 9, 11], contrasting with the spherical, cristae-poor mitochondria of the inner cell mass and embryonic stem cells [16]. Microarray analyses have detected differences in the expression of genes regulating mitochondria between human iPS cells and ES cells [7, 8, 17, 18]. In addition, Prigione et al [19] reported the acquisition of mutations in human iPS cell mtDNA not present in the parental cells. Inconsistencies between ES cell and iPS cell metabolism could be explained by incomplete or inappropriate reprogramming of metabolism during iPS cell formation, which links directly with the modulation of epigenetic mechanisms, and raises questions of how reliably metabolism is reprogrammed during this process.

The metabolic profiles of human ES cells in culture are relatively well characterized, as are the metabolic changes that these cells undergo in response to oxygen, a well characterized regulator of cell physiology and gene expression. In comparison to cells cultured in ambient (20%) oxygen, human ES cells increase glucose consumption and lactate production under physiological (5%) oxygen [20–23], accompanied by increased total amino acid turnover [21, 22, 24]. These changes are mediated via Hypoxia-Inducible Factor 2 activity [25]. Oxygen is similarly a significant physiological regulator of preimplantation embryo development, which displays an ability to modulate carbohydrate and amino acid metabolism in response to oxygen [26, 27]. Well documented negative effects of atmospheric oxygen on development to the blastocyst, cell number, apoptosis, gene expression, global DNA methylation, the proteome, and ultimately embryo viability [28–35], support a regulatory role for physiological oxygen conditions during development.

In contrast to ES cells, the responses of iPS cells to environmental challenge, and specifically to culture under physiological oxygen conditions, have not been extensively studied. Therefore, the aim of this study was to compare the metabolism of three independently derived human iPS cell lines cultured in 5% and 20% oxygen to determine whether the reprogrammed metabolism of these cells was consistent with the metabolism of an embryo-derived (ES) cell line, and whether the metabolism of iPS cells was able to respond to physiological challenge. Metabolic profiles of human iPS cells were estimated using ^1^H-NMR spectroscopy of medium metabolites in spent culture medium and compared to a control human ES cell line. Only one of the human iPS cell lines tested modulated carbohydrate metabolism, as did the ES cell line, in response to changing oxygen concentration. Significantly, the oxygen responsive human iPS cell line was easily discriminated from the human ES cell line and the other iPS cell lines using partial least squares discriminant analysis (PLSDA), indicating that it differed in other metabolic pathways. These findings infer that the metabolism of the iPS cell lines examined has not been functionally reprogrammed and does not recapitulate the metabolic responsiveness of ES cells, and emphasise the need to develop reprogramming methods that promote a normal and responsive underlying cellular physiology.

## Materials and methods

### Pluripotent stem cell culture

MEL1 human ES cells [36], PDL-D1C6 (PDL [37]), NHF1.3 [38], and IMR90 iPS cells [39] were used in this study. PDL iPS cells were generated by lentiviral transduction of human periodontal ligament fibroblasts using the four Yamanaka factors (OCT4, SOX2, KLF4 and cMyc; [40]). NHF1.3 iPS cells were generated from human fibroblasts using an episomal vector based strategy using the four Yamanaka factors. IMR90 iPS cells were generated by retroviral transduction of OCT4, SOX2, KLF4 and LIN28 into human fetal lung fibroblasts. All research performed on human cell lines was approved by the CSIRO Ethics Committee and The University of Melbourne Ethics Committee.

Cell lines were transferred from their original culture conditions to mTeSR1^™^ medium (Stem Cell Technologies, Vancouver, Canada) [41] on ES-cell qualified Matrigel^™^ (Corning Life Sciences, Tewksbury, MA, USA)–coated tissue culture plates (Corning Life Sciences) and maintained at 37°C in an atmosphere of 5% CO_2_ in a humidified Forma CO_2_ Steri-Cycle incubator (Thermo Scientific, Scoresby, VIC, Australia). Cells were passaged every 5–7 days using Dispase (Stem Cell Technologies) and were cultured in mTeSR1^™^ for a minimum of 4 passages under ambient oxygen conditions prior to experimentation. To assess oxygen responsiveness cells were sub-cultured into paired plates that were maintained at 37°C in an atmosphere of either 20% oxygen as outlined above, or 5% oxygen in a Forma tri-gas Steri-Cycle incubator (Thermo Scientific). Medium was pre-equilibrated under the respective oxygen condition and used to replace the culture medium every 24 hours. All cell cultures were acclimated to 5% or 20% oxygen conditions for a minimum of 2 passages before collection and analysis. At subsequent passages, concurrently seeded wells of cells were used to establish cultures in Falcon 12-well tissue culture plates (Becton Dickinson). Precisely 24 hours after medium replenishment on day 4 after passage, spent medium was collected, centrifuged to remove cellar debris, transferred to rubber o-ring vials (Jet Biofil, Bengaluru, Karnataka, India) to prevent gas exchange and stored at -80°C until metabolite extraction. Cell numbers were determined by generating a single cell suspension using TrypLE Select (Life Technologies, Scoresby, VIC, Australia) and counting on a hemocytometer. Two wells from each of the 5% and 20% oxygen plates were chosen for NMR analysis, based on the similarity of total cell numbers, and the experiments were repeated 4 times. One well coated with ES-cell qualified Matrigel^™^, containing medium only, was used as an unspent medium control. Spent medium samples were examined from the following passage ranges: MEL1 human ES cells: p41-46; PDL iPS cells: p27-32; NHF1.3 iPS cells: p69-74; and IMR90 iPS cells: p40-45.

The use of a fully defined, commercially available medium (mTeSR1^™^) that maintains a largely undifferentiated pluripotent cell population enabled accurate determination of the effect of oxygen on metabolic characteristics and eliminated confounding factors, such as feeders, serum and high levels of differentiation.

### Fibroblast cell culture

PDL fibroblasts were maintained in α-minimal essential medium (α-MEM; Sigma-Aldrich) containing 10% fetal calf serum (FCS; Hyclone Laboratories, Logan, UT), 2 mm L-glutamine (Sigma-Aldrich), 100 μM L-ascorbate-2-phosphate (Sigma-Aldrich), 1 mM sodium pyruvate (Sigma-Aldrich), 50 U/mL penicillin G and 50 μg/mL streptomycin (Sigma Aldrich) [37]. IMR90 fibroblasts were cultured in Eagle’s Minimum Essential Medium (Sigma-Aldrich) supplemented with 10% fetal bovine serum (FBS; HyClone), 0.1 mM non-essential amino acids (Sigma-Aldrich), 1.0 mM sodium pyruvate (Invitrogen) and 1X Pen/Strep (Invitrogen) [39]. Media were refreshed every 48h for both cell lines.

### Spent medium metabolite extraction for NMR spectroscopy

For the determination of human ES and iPS cell metabolite use by NMR spectroscopy, metabolite extraction was performed on 180 μL of medium sample as described previously [22]. Briefly, each sample was spiked with 20 μL of 5 mmol/L imidazole in deuterium oxide (D_2_O) as an internal standard for metabolite recovery. Samples were diluted with ice-chilled methanol-d_4_ in a 2:1 ratio. Samples were rested on ice for 15 min, centrifuged (10000*g*, 21°C, 15 min) and 270 μL of protein-free supernatant (containing 90 μL spiked medium) was recovered. Subsequently, 270 μL (200 mmol/L) of sodium phosphate in D_2_O (buffered to pH 7 using deuterium chloride) was added to each sample along with a further 60 μL of D_2_O containing 5 mmol/L 2,2-dimethyl-2-silapentane-5-sulfonic acid-d_6_ sodium salt (DSS) as an NMR reference compound and 0.2% w/v sodium azide (final sample volume of 600 μL). Prepared samples were loaded into NMR tubes (7 inch, 507 grade with a 5 mm diameter; Wilmad LabGlass, Vineland, NJ, USA) for subsequent spectroscopic analysis.

### NMR spectroscopy, spectral identification and metabolite quantification

Samples were analysed on a 600MHz NMR spectrometer (Bruker Biospin, Alexandria, NSW, Australia) and quantified according to the procedure outlined in Sheedy *et al*. [42]. The following parameters were used: NOSEY 1D pulse sequence (90° pulse width = 20 μs, d1 = 1.5 s, d8 = 0.5 s, d11 = 0.03 s, d12 = 0.00002 s), 256 scans, sample temperature = 25°C, sweep width = 12 ppm. Automatic Exponential Fourier transformation and phase correction was performed using Topspin 3.1 (Bruker) followed by amplitude normalization of all spectra to the internal standard (DSS). Signals were identified and quantified using Chenomx NMR Suite software (Chenomx, Edmonton, Alberta, Canada). Validation of peak identity was performed by a 2D TOCSY NMR experiment as described previously [43].

Logarithm (log_10_) transformation and median normalization was applied to a data matrix of quantified metabolites to centre and scale prior to multivariate data analysis as previously described [43]. To visualize the cluster pattern of the data set, PCA and PLSDA were then applied to the data using Unscrambler X software (Camo, Oslo, Norway). Quantified amino acids, glucose and lactate were normalized to imidazole to correct for variation in metabolite recovery, and to cell number. Control values (spent medium without cells) were subtracted from their matching samples to give consumption and production values. Amino acid turnover was calculated as the sum of amino acids consumed and produced. Glycolytic rate was calculated on the assumption that one mole of glucose would give rise to 2 moles of lactate, where percentage glycolysis = (# moles of lactate) / (# moles of glucose x2).

### Analysis of fibroblast carbohydrate utilisation

PDL and IMR90 fibroblasts were cultured as described under either 5% or 20% oxygen for a minimum of 2 passages prior to analysis. For analysis, cells were seeded into 12-well plates and cultured for 96h. A medium only control lacking cells was also maintained. Spent medium samples (n = 3 independent biological replicates over 3 passages) were collected from each well 48 hours after medium renewal, snap-frozen in liquid nitrogen and stored at -80°C. Cells were then reduced to a single-cell suspension using trypsin EDTA (Invitrogen) and counted on a haemocytometer.

Glucose consumption was determined by the reduction of nicotinamide adenine dinucleotide phosphate (NADP+) by glucose 6-phosphate dehydrogenase to NADPH as described previously [21]. Lactate production was determined through the reduction of nicotinamide adenine dinucleotide (NAD+) to NADH by lactate dehydrogenase. Collected media samples were added to a glucose assay (3.7 mM MgSO4.7H2O, 0.6 mM NADP+, 0.5 mM ATP, 0.5 mM dithiothreitol, 12 U hexokinase/mL, and 6 U G6PDH/mL in EPPS buffer, pH 8.0; [44]) and to a lactate cocktail (4.76 mM NAD+, 100 U LDH/mL, and 2.6 mM EDTA in glycine-hydrazine buffer, pH 9.4; (Gardner and Leese 1990) and incubated for 30 minutes. Following incubation, the fluorescence of NADPH or NADH produced was quantified fluorometrically, respectively. The concentration of glucose and lactate in the spent medium samples was determined from a standard curve generated from known concentrations of glucose or lactate respectively.

### Statistical analyses

All data were analysed for normality and homogeneity of variance using SPSS Statistics (IBM Corporation, Armonk, NY) and GraphPad Prism (GraphPad Software, La Jolla CA). Metabolite data were analysed by ANOVA with Bonferroni post hoc analysis, or non-parametrically by Kruskal-Wallis with Dunn’s comparison using SPSS Statistics and GraphPad Prism. Fibroblast carbohydrate use data were analysed by unpaired t-tests. PLS-DA, for the 2 principal factors accounting for the greatest amount of covariance in the quantified data matrix of metabolites, were used to create 2D scores plots (representing the relationship between samples) and loadings plots (representing the relationship between metabolites). Each factor modelling the greatest amount of covariance within the data matrix of metabolites was plotted orthogonally to each other, to provide a model for visual interpretation of relationships in a data set with high-dimensionality [43]. Results were considered statistically significant at *P* < 0.05. All data are presented as mean ± SEM.

## Results

### Pluripotent stem cells display distinct metabolite profiles

**Fig 1:**
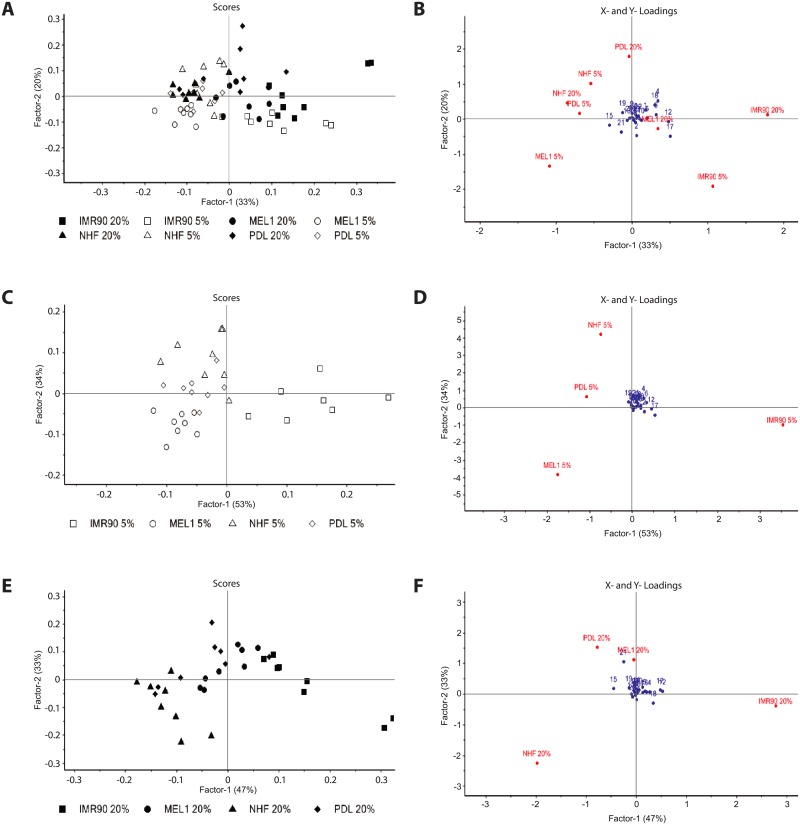
Partial Least Squares Discriminant Analysis (PLSDA) of metabolite concentrations present in spent medium from IMR90, PDL and NHF1.3 human iPS cells and MEL1 human ES cells. (**A, C, E**) PLSDA scores plots of medium samples from human ES and iPS cells cultured in 5% and 20% oxygen (A), 5% only (C) and 20% only (E). Each data point represents the entire metabolite profile for each sample. Samples closer together have similar metabolite profiles; samples diagonally opposed are more dissimilar. (**B, D, F**) PLSDA loadings plots of metabolite use by human ES and iPS cells cultured in 5% and 20% oxygen (B), 5% only (D) and 20% only (F). n = 8 samples per line per treatment, from 4 independent biological replicates. Numbers refer to metabolites: (1) alanine, (2) arginine, (3) aspartate, (4) cystine, (5) GABA, (6) glucose, (7) glutamate, (8) glutamine, (9) glycine, (10) histidine, (11) isoleucine, (12) lactate, (13) leucine, (14) lysine, (15) methionine, (16) phenylalanine, (17) proline, (18) pyruvate, (19) serine, (20) threonine, (21) tryptophan, (22) tyrosine, (23) valine.

Metabolite production and consumption by the three iPS cell lines and a control human ES cell line were profiled using ^1^H-NMR spectroscopy. Partial Least Squares Discriminant Analysis (PLSDA) of metabolite levels from all cell lines revealed distinct metabolic profiles across cell lines, with a marked difference in metabolite use in response to oxygen displayed by MEL1 human ES cells and IMR90 iPS cells (Fig 1A and 1B). In contrast, the overall metabolite profile of NHF1.3 iPS and PDL iPS cells appeared to be unaffected by a reduction in oxygen concentration from 20% to 5%. The analysis of metabolite levels of lines cultured under either 5% (Fig 1C and 1D) or 20% (Fig 1E and 1F) oxygen showed a similar discrimination based on cell line, where the overall metabolite profile of IMR90 iPS cells was distinct compared with that of NHF1.3 and PDL iPS cells, and MEL1 human ES cells.

### Amino acid use and turnover discriminates individual iPS cell lines

**Fig 2:**
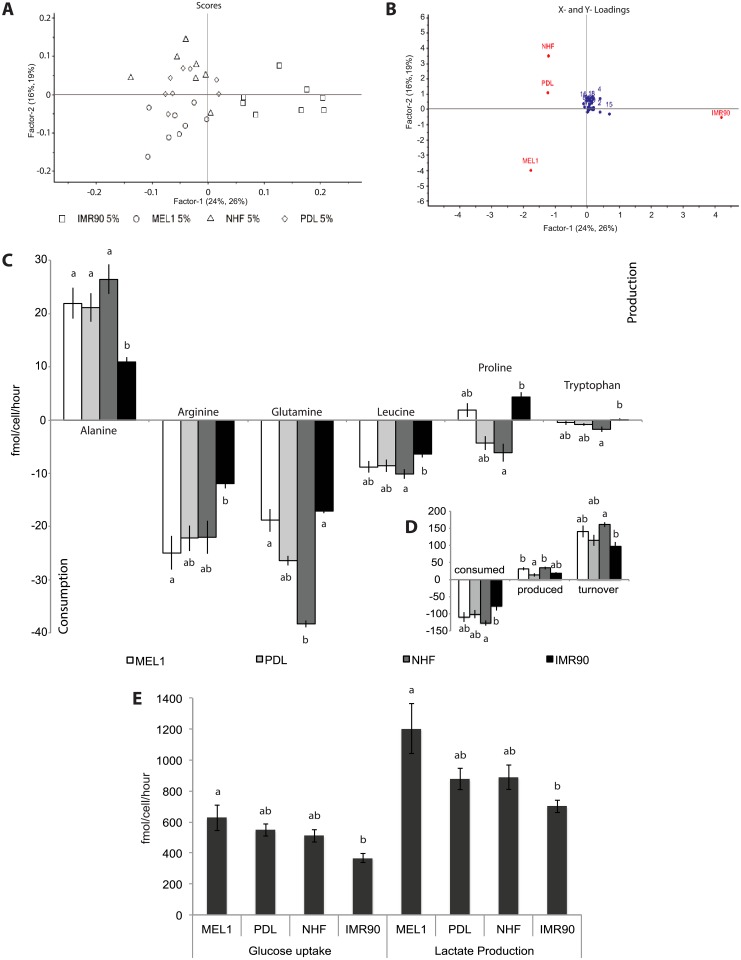
Comparison of metabolite use by cell lines cultured under 5% oxygen conditions. (**A**) PLSDA scores plot of amino acid concentrations of human ES and iPS cells cultured in mTeSR medium under 5% oxygen. (**B**) PLSDA loadings plot of amino acid concentrations of human embryonic and iPS cells cultured in mTeSR medium under 5% oxygen. (**C**) Significant differences in amino acid utilisation, (**D**) total amino acid consumption, production and turnover, and (**E**) glucose uptake and lactate production between MEL1 human ES, PDL, NHF1.3 and IMR90 iPS cells cultured under 5% oxygen. Metabolites were quantified in spent medium samples following a 24-hour incubation period (day 4–5) by ^1^H-NMR, and normalised to cell number and an internal standard (imidazole). Data are presented as mean ± SEM; n = 8 samples per line per treatment. ^a,b^ Different superscripts represent statistically significant differences within a metabolite, production, consumption or total amino acid turnover; P<0.05. Numbers refer to metabolites: (1) alanine, (2) arginine, (3) aspartate, (4) cystine, (5) GABA, (6) glucose, (7) glutamate, (8) glutamine, (9) glycine, (10) histidine, (11) isoleucine, (12) lactate, (13) leucine, (14) lysine, (15) methionine, (16) phenylalanine, (17) proline, (18) pyruvate, (19) serine, (20) threonine, (21) tryptophan, (22) tyrosine, (23) valine.

Amino acid use, in cells cultured under reduced (5%) oxygen, discriminated between cell lines (Fig 2A and 2B). Under 5% oxygen, alanine production and arginine consumption were significantly reduced in IMR90 iPS cells (P = 0.0004 and P = 0.012 respectively; Fig 2C). In contrast to the production of proline by human ES cells in culture [21–24, 45], proline use by human pluripotent stem cell lines cultured under 5% oxygen was variable in the current study, with proline being produced by MEL1 ES cells and IMR90 iPS cells, while being consumed by NHF1.3 and PDL iPS cells (P = 0.013; Fig 2C). In addition, the use of leucine and tryptophan was significantly altered in IMR90 iPS cells cultured under 5% oxygen, compared with NHF1.3 iPS cells (Fig 2C, P = 0.05 and P = 0.025 respectively). Glutamine consumption discriminated between NHF1.3 iPS cells and both MEL1 ES cells and IMR90 iPS cells, with consumption being significantly higher in NHF1.3 iPS cells at 5% oxygen (P = 0.0048; Fig 2C).

**Fig 3:**
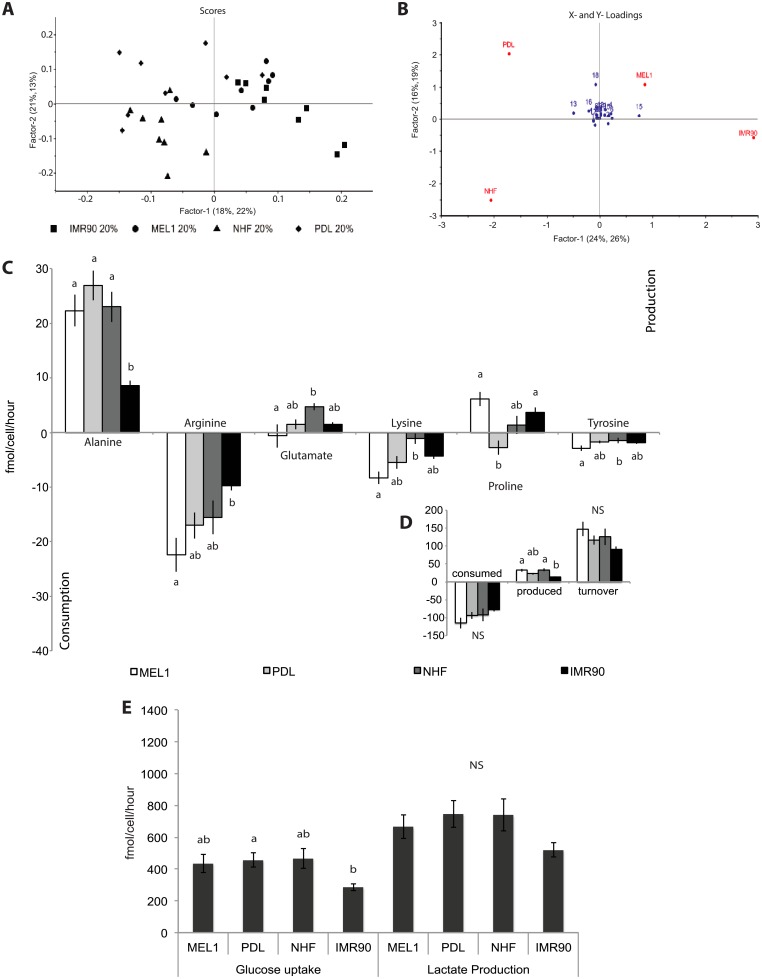
Comparison of metabolite use between cell lines cultured under 20% oxygen conditions. (**A**) PLSDA scores plot of amino acid concentrations of human embryonic and iPS cells cultured in mTeSR medium under 20% oxygen. (**B**) PLSDA loadings plot of amino acid concentrations of human embryonic and iPS cells cultured in mTeSR medium under 20% oxygen. (**C**) Significant differences in amino acid utilisation, (**D**) total amino acid consumption, production and turnover, and (**E**) glucose uptake and lactate production between MEL1 human ES, PDL, NHF1.3 and IMR90 iPS cells cultured under 20% oxygen. Metabolites were quantified in spent medium samples following a 24 hour incubation period (day 4–5) by ^1^H-NMR, and normalised to cell number and an internal standard (imidazole). Data are presented as mean ± SEM; n = 8 samples per line per treatment. ^a,b^ Different superscripts represent statistically significant differences within a metabolite, production, consumption or total amino acid turnover; P<0.05. NS = not significantly different. Numbers refer to metabolites: (1) alanine, (2) arginine, (3) aspartate, (4) cystine, (5) GABA, (6) glucose, (7) glutamate, (8) glutamine, (9) glycine, (10) histidine, (11) isoleucine, (12) lactate, (13) leucine, (14) lysine, (15) methionine, (16) phenylalanine, (17) proline, (18) pyruvate, (19) serine, (20) threonine, (21) tryptophan, (22) tyrosine, (23) valine.

Quantitation of amino acid use by cells cultured under 20% oxygen also discriminated between the cell lines (Fig 3A and 3B). Similar to cells cultured under 5% oxygen, alanine and arginine were able to discriminate IMR90 iPS cells cultured under 20% oxygen (P = 0.0004 and 0.007 respectively; Fig 3C). Additionally, proline was consumed by PDL iPS cells under 20% oxygen, while it was produced in MEL1 ES cells and NHF1.3 and IMR90 iPS cells (P = 0.002; Fig 3C). The use of glutamate (P = 0.014), lysine (P = 0.001) and tyrosine (P = 0.05) also discriminated between MEL1 ES cells and NHF1.3 iPS cells at 20% oxygen (Fig 3C).

Independent of oxygen concentration, total amino acid consumption and turnover by IMR90 iPS cells was less than that observed for MEL1 ES cells and NHF1.3 iPS cells (P = 0.02, P = 0.001 and P = 0.001 respectively; Figure A in S1 File). Similarly, total amino acid production was significantly different between lines, independent of oxygen treatment (P<0.001), where both PDL and IMR90 iPS cells differed significantly from MEL1 human ES and NHF1.3 iPS cells (Figure A in S1 File).

Under 5% oxygen conditions, total amino acid production by MEL1 human ES and NHF1.3 iPS cells was significantly higher than PDL iPS cells (P = 0.0022, Fig 2D). Total amino acid consumption by IMR90 iPS cells was significantly lower than that of NHF1.3 iPS cells, but not significantly different from MEL1 human ES or PDL iPS cells (P = 0.024; Fig 2D). Similarly, total amino acid turnover by IMR90 iPS cells was significantly lower than that of NHF1.3 iPS cells, but not significantly different from MEL1 human ES or PDL iPS cells (P = 0.005; Fig 2D). Neither total amino acid consumption nor total amino acid turnover differed significantly between lines under 20% oxygen conditions (Fig 3D), however total amino acid production was significantly higher in MEL1 human ES and NHF1.3 iPS cells compared with IMR90 iPS cells (P = 0.0001; Fig 3D).

### Oxygen regulated carbohydrate use delineates between high and low metabolic fidelity in human pluripotent stem cell lines

Following culture under 5% oxygen, glucose uptake and lactate production were significantly lower in IMR90 iPS cells when compared with MEL1 human ES cells (P = 0.012 and 0.026 respectively; Fig 2E). Comparison of cell lines cultured under 20% oxygen found only glucose uptake by PDL iPS cells to be significantly higher than that observed in IMR90 iPS cells (P = 0.021; Fig 3E), with no other significant differences in lactate production between lines apparent. Independent of oxygen concentration, IMR90 iPS cells consumed less glucose (P = 0.001) and produced less lactate (P<0.001) than either PDL or NHF iPS cells (Figure B in S1 File).

**Fig 4:**
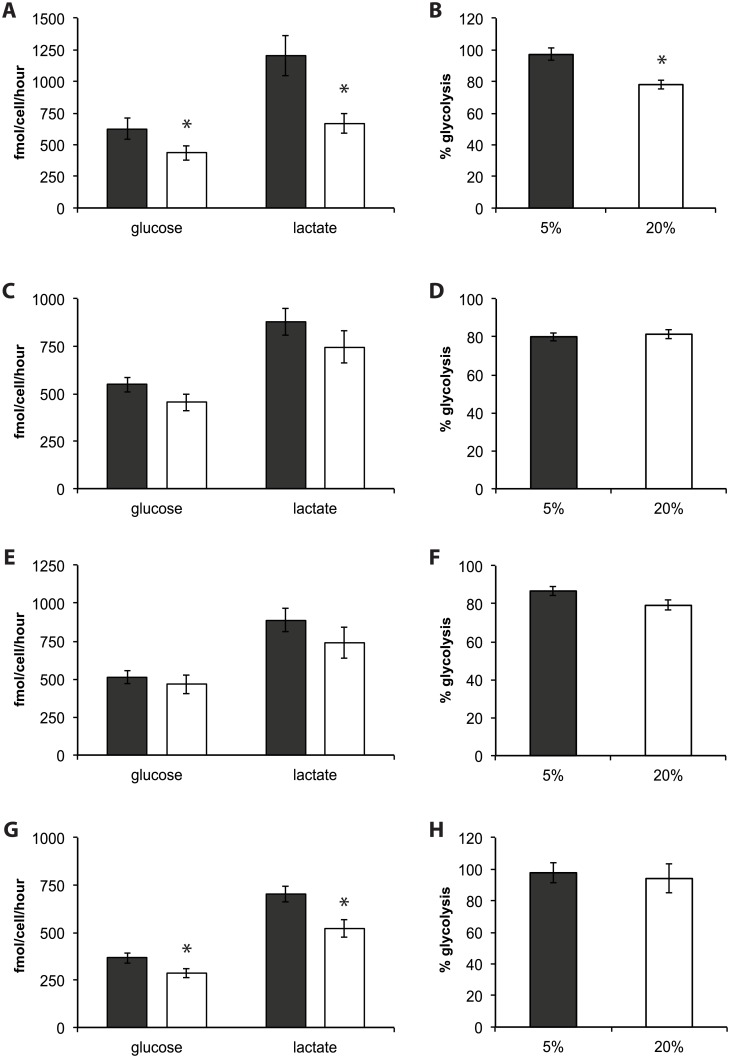
Carbohydrate utilisation and percentage glycolysis in MEL1 human ES (A, B), PDL (C, D), NHF1.3 (E, F) and IMR90 (G, H) human iPS cells in response to oxygen. (**A, C, E, G**) Glucose consumption and lactate production, quantified in spent medium samples by ^1^H-NMR, normalised to cell number and an internal standard (imidazole). (**B, D, F, H**) Glycolytic rate (% glycolysis) for each cell line was calculated as the number of moles of lactate / 2 x number of moles of glucose. Black bars: 5% oxygen; white bars: 20% oxygen. Data are presented as mean ± SEM; n = 8 samples per line per treatment. * P<0.05, ** P<0.01.

Culture at 5% oxygen has been shown previously to increase the flux of glucose and lactate measured in human pluripotent cells [20, 21]. A significant increase in glucose flux to lactate was seen when MEL1 human ES cells (P = 0.01) and IMR90 iPS cells (P = 0.02) were transferred to an atmosphere containing 5% oxygen (Fig 4A and 4G respectively). No difference in glucose consumption or lactate production by PDL or NHF1.3 iPS cells was observed in response to oxygen (Fig 4C and 4E respectively, P>0.05). When expressed as the conversion of 1 mole of glucose to 2 moles of lactate, glycolytic flux was significantly altered by oxygen in MEL1 human ES cells (Fig 4B), but was not statistically different between oxygen treatments for any iPS cell line (Fig 4D, 4F and 4H, P>0.05).

To examine metabolic regulation of the parental fibroblasts in response to oxygen, carbohydrate use was determined for PDL and IMR90 fibroblasts cultured under 5% and 20% oxygen conditions. Exposure of PDL fibroblasts to physiological oxygen conditions did not alter glucose consumption or lactate production (P>0.05; Figure A and Figure C in S2 File, respectively). In contrast, physiological oxygen culture significantly altered carbohydrate use in IMR90 fibroblasts. Culture under 5% oxygen significant increased glucose use by IMR90 fibroblasts (P = 0.002; Figure B in S2 File), while it significantly reduced lactate production (P = 0.01; Figure D in S2 File).

### Oxygen modulates individual amino acid use in human iPS cells

**Fig 5:**
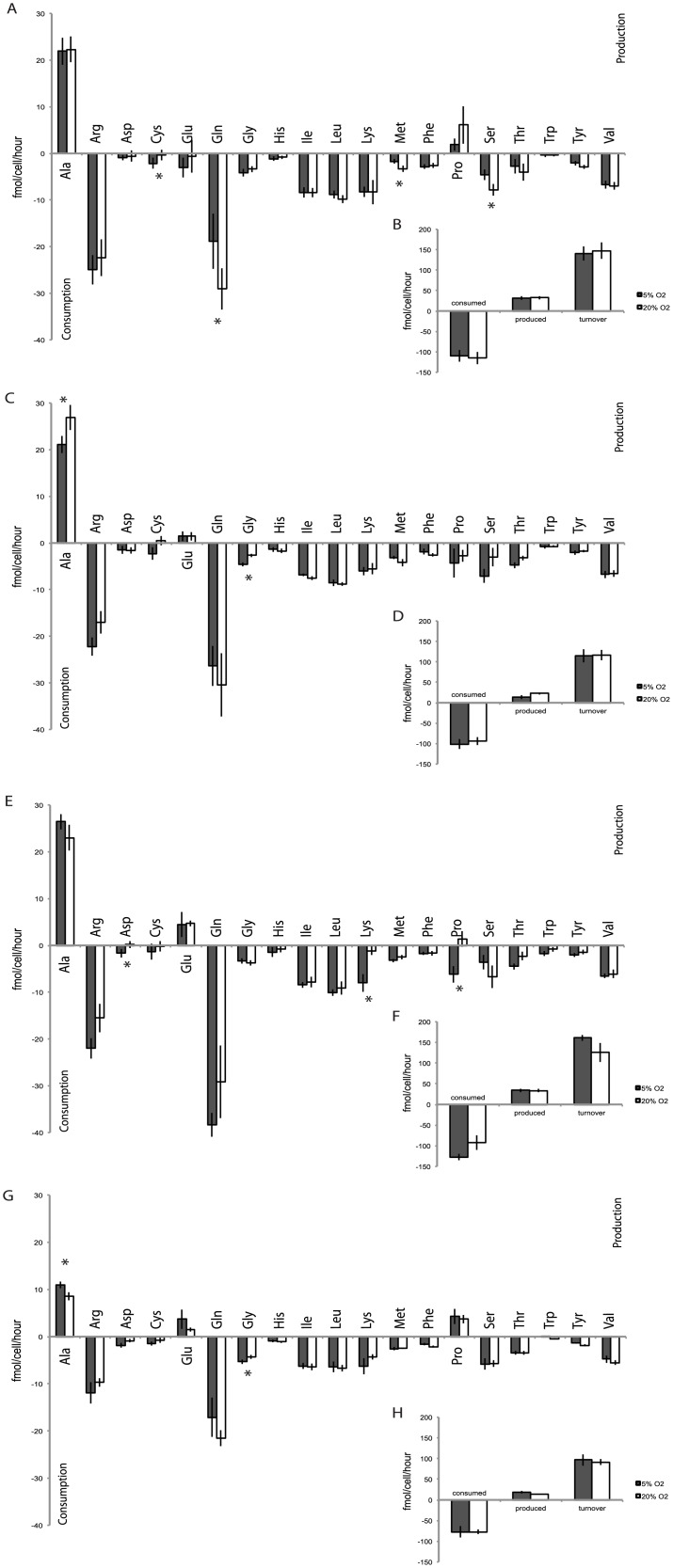
Amino acid utilisation profiles in MEL1 human ES (A, B), PDL (C, D), NHF1.3 (E, F) and IMR90 (G, H) human iPS cells in response to oxygen. Spent media samples were collected after a 24-hour incubation period (day 4–5), normalised to cell number and an internal standard (imidazole) for analysis by ^1^H-NMR. Black bars: 5% oxygen; white bars: 20% oxygen. (**A, C, E, G**) Individual amino acid production and consumption. (**B, D, F, H**) Total amino acid production, consumption and turnover. Data are presented as mean ± SEM; n = 8 samples per line per treatment. * P<0.05, ** P<0.01.

Oxygen did not consistently regulate specific amino acids across all lines examined. MEL1 human ES cell glutamine (P = 0.02), methionine (P = 0.02) and serine (P = 0.03) consumption were significantly higher under 20% compared to 5% oxygen, while cysteine consumption was higher in MEL1 human ES cells cultured under 5% oxygen (P = 0.03; Fig 5A). Individual amino acid use by PDL iPS cells was largely unaffected by oxygen, with only increased alanine production and decreased glycine consumption observed in cells cultured under 20% compared to 5% oxygen (P = 0.01, Fig 5C). Oxygen concentration significantly altered the utilization of aspartate (P = 0.05), lysine (P<0.001) and proline (P<0.001) levels in NHF1.3 iPS cells. Lysine consumption was significantly lower following 20% oxygen culture. Both asparatate and proline were consumed under 5% oxygen, while they were produced under 20% oxygen (Fig 5E). Similar to PDL iPS cells, alanine production was higher in IMR90 iPS cells cultured under 20% oxygen compared with those cultured under 5% (P = 0.006), while glycine consumption was higher under 5% oxygen culture compared with 20% oxygen (P = 0.03; Fig 5G). Atmospheric oxygen did not significantly alter total amino acid production, consumption or turnover in any pluripotent stem cell line compared with those cultured at 5% oxygen (Fig 5B, 5D, 5F and 5H respectively).

## Discussion

Historically, the analysis of iPS cell lines has focused on the characterization of pluripotency and differentiation potential, genomic integrity and the degree to which these cells do or do not retain characteristics of their somatic progenitors. Such analyses have shown significant variability between iPS cell lines at the level of transcription and within the epigenome [12, 46–48]. The realization that metabolic remodelling plays a key role in the reprogramming process [1] raises similar questions around the consistency of metabolic reprogramming and the metabolic fidelity of reprogrammed cells. Here we examined metabolic properties of three independently derived human iPS cell lines and assessed the ability of these cell lines to modulate metabolism in response to oxygen, a known regulator of the embryonic and pluripotent metabolome [20–24, 26, 27]. The iPS cell lines examined were metabolically distinct from the control human ES cell line, and failed to display equivalent, or characteristic responses to changes in extracellular oxygen concentration. Results of the present study suggest that these iPS cell lines fail to acquire metabolic fidelity, defined by an inability to regulate their metabolism in response to oxygen, plausibly as a result of metabolic memory and incomplete or aberrant metabolic programming during the reprogramming process.

### Reprogrammed cells display a metabolic memory

The human iPS cell lines examined in the current study displayed high levels of glycolysis, in the range of 70–80%, a feature of human ES cell lines in culture [2, 8, 21, 22, 45] and the MEL1 ES cell line examined, which likely reflects an acquired dependence on glycolysis for biosynthesis. However, in contrast to human ES cells, the preimplantation embryo, and the majority of cell types, NHF1.3 and PDL iPS cell lines failed to regulate glucose uptake and lactate production in response to changes in the extracellular oxygen concentration. Significantly, the inability of PDL iPS cells to respond to oxygen appears to reflect a somatic metabolic memory, as the parent fibroblasts likewise did not regulate carbohydrate metabolism in response to oxygen challenge. Conversely, the ability of IMR90 iPS cells to regulate carbohydrate metabolism plausibly reflects oxygen responsiveness in the parental fibroblasts, although the regulation of lactate production was inverse to that reported for derived iPS cells, suggesting a partial restructuring of metabolism. Data similarly suggest that while IMR90 iPS cells display regulation of carbohydrate use, they display a distinct metabolic phenotype, and that the activity of other metabolic pathways discriminates these cells from human ES cells and the other iPS cell lines. Hence, these data suggest that metabolism can discriminate between iPS cell lines, and that the metabolism of all iPS cell lines is not equivalent to that of human ES cells.

Folmes et al [49] discriminated between mouse iPS cell lines based on acetate, lactate and glucose metabolism, showing that iPS cells generated using cMyc consumed more glucose and produced more lactate and acetate, than those generated in the absence of cMyc. Although the lines examined in the present study were derived differently, the IMR90 iPS cells were singular in that they were generated in the absence of cMyc [39] and showed lower glucose consumption and lactate production and a metabolism distinct from the other cell lines examined. Inclusion of cMyc is known to repress fibroblast-specific gene expression while enhancing metabolic gene expression during the early phase of reprogramming [50]. Plausibly, the absence of cMyc during reprogramming hinders the acquisition of a more glycolytic phenotype. Analysis of additional reprogrammed cell lines will be necessary to confirm the impact of cMyc on metabolic reprogramming, as well as determine whether other reprogramming factors, and/or methods, result in complete metabolic reprogramming.

While this study was limited to the assessment of 3 iPS cell lines, derived using different parental cell types and methods of generation, findings highlight the inability of standard metabolic profiling in isolation to reveal key physiological differences. Additional metabolic analyses, and the inclusion of additional lines, would further elucidate the extent of metabolic memory retention by iPS cells. Recently, Park et al [51] reported significant differences in mitochondrial activity and specific metabolite levels in partially reprogrammed iPS cells, relative to fully reprogrammed iPS cells. In addition, Prigione et al [19] documented the acquisition of mtDNA mutations in human iPS cell lines not present in the parental cell line following reprogramming, while mouse iPS cells show altered mitochondrial replication upon differentiation relative to ES cells [10]. These data suggest that mitochondria may be particularly sensitive during the reprogramming process. Whether the inability to regulate metabolism in response to oxygen reflects compromised mitochondrial regulation remains to be determined.

In addition to the retention of a somatic metabolic memory, the lack of acquisition of embryonic stem cell-like metabolic fidelity in iPS cells may arise from cell age, the culture conditions or practices used, or alternatively, may become established during the reprogramming process itself, independent of the acquisition of pluripotency. Nevertheless, as glucose-derived pyruvate has been shown to be important in modulating acetyl-CoA levels in human ES cells, required for the maintenance of histone acetylation [52], altered glucose flux, mitochondrial activity and metabolic adaptation and plasticity by iPS cells will plausibly lead to altered epigenetic dynamics.

### Perturbations in metabolite use may alter cell viability

Physiological oxygen resulted in varying amino acid regulation in the current study. Differences in alanine and glycine utilization were evident in response to oxygen in IMR90 and PDL iPS cells, while NHF1.3 iPS cells regulated aspartate, lysine and proline use in response to oxygen. More significantly, while oxygen did not elicit consistent changes in specific amino acids across the pluripotent stem cell lines studied, analysis of amino acid turnover highlighted cell line specific perturbations in alanine, glycine and proline production and consumption. Pronounced differences in amino acid metabolism have been documented previously in mouse iPS cells relative to ES cells [53]. In the current study, a change in proline use from production to consumption was observed in PDL iPS cells, irrespective of oxygen concentration, as well as in NHF1.3 iPS cells cultured under 5% oxygen conditions, relative to both MEL1 human ES cells and IMR90 iPS cells examined in this study, and to documented profiles in other ES cell lines [21–24]. Alterations in proline use have been documented in response to the presence of KOSR in human ES cell culture medium [45]. More significantly, proline is the only amino acid whose availability has been associated with the induction of differentiation of ES cells [54, 55], accompanied by changes to replication timing [56]. The ramifications of even subtle changes in amino acid use on human pluripotent stem cell physiology, and more significantly their differentiated derivatives, remain unclear, but likely impact the epigenome [57], as has been observed with the provision or exclusion of glutamine [58] and threonine [59], and the presence of proline [56] in mouse ES cell cultures, and in the absence of methionine in human ES cell culture [60]. Plausibly, altered amino acid use reflects an altered cell state.

### Implications of loss of metabolic fidelity for human iPS cell generation

While alterations in metabolism do not appear to alter the maintenance of self-renewal within the iPS cells examined per se [37–39], remodelling of cellular metabolism may alter other aspects of cell function. The importance of appropriate metabolic control is well documented in preimplantation embryos, as well as numerous diseases. Perturbations in carbohydrate and amino acid metabolism have been shown to affect the pre- and post-implantation viability and differentiation of mammalian blastocysts [32, 61, 62]. Therefore, while pluripotent stem cell self-renewal may not be adversely affected by alterations in metabolism, such plasticity could have significant functional consequences for differentiated derivatives, or may impact differentiation kinetics.

As methods of iPS cell generation evolve there is a need to establish whether cell physiology and acquisition of a responsive pluripotent stem cell metabolic profile is achieved, along with improvements in efficiency. Establishment of metabolic fidelity in iPS cells, equivalent to that of ES cells, will be particularly important for iPS models of disease where characterization of disease aetiology, as well as utility for drug discovery, is reliant on an accurate manifestation of disease. Modelling diseases will require not only detailed characterization of multiple cell lines derived from multiple patients, but our data reveal the need to assess whether the cellular phenotypes of these cells is truly representative of the disease irrespective of whether the disease has a metabolic phenotype. Significantly, metabolic profiling in isolation is insufficient to delineate the metabolic fidelity of pluripotent stem cells. Rather, examination of metabolism in response to physiological challenge both prior to and following differentiation will be necessary to ensure the metabolic stability of these cells.

## Conclusions

It is clear that reprogramming of somatic cells to an embryonic-stem cell like state can result in significantly altered metabolite profiles without impacting the acquisition of self-renewal. Unlike previous reports that document baseline metabolite profiles under equivalent culture conditions, it is apparent from this study that not only do overall profiles differ between pluripotent cell types, but the ability of reprogrammed iPS cell lines to modulate metabolism in response to a physiological stimulus in culture can be impaired. Data indicate the retention of a parental fibroblast metabolic response to oxygen. If the clinical and commercial potential of human iPS cells is to be reached, a greater understanding of metabolic activity and the regulation of metabolic processes during iPS cell generation and differentiation will need to be developed. The ability of pluripotent stem cell lines to establish an appropriate physiology with different methods of generation, and from different starting cell types, particularly with respect to their ability to respond to stimuli, requires further investigation. This may also involve critical development of culture medium formulations that support the appropriate reprogramming of metabolism. Differences between iPS cells and ES cells documented in the current study, and previously, highlight the need to incorporate more exhaustive evaluations of both pluripotent stem cell sources, particularly at a physiological level, before the utility of these cells in research, disease models and drug testing can be realized.

## Supporting information

S1 Fig Metabolite utilisation by MEL1 human ES, PDL, NHF1.3 and IMR90 human iPS cells independent of oxygen. **(A)** Total amino acid production, consumption and turnover. **(B)** Glucose consumption and lactate production. Spent media samples were collected after a 24-hour incubation period (day 4–5 of passage), normalised to cell number and an internal standard (imidazole) for analysis by ^1^H-NMR. Data are presented as mean ± SEM. ^a,b^ Different superscripts represent statistically significant differences within a metabolite, production, consumption or total amino acid turnover; P≤0.003.
(EPS)

S2 Fig Carbohydrate utilisation in PDL (A, B) and IMR90 (C, D) fibroblasts in response to oxygen. (**A, C**) Glucose consumption, quantified in spent medium samples, normalised to cell number and an unspent medium control. (**B, D**) Lactate production, quantified in spent medium samples, normalised to cell number and an unspent medium control Black bars: 5% oxygen; white bars: 20% oxygen. Data are presented as mean ± SEM; n = 3 samples per line per treatment. ** P = 0.01, *** P = 0.002.
(EPS)
